# How visual search relates to visual diagnostic performance: a narrative systematic review of eye-tracking research in radiology

**DOI:** 10.1007/s10459-016-9698-1

**Published:** 2016-07-19

**Authors:** A. van der Gijp, C. J. Ravesloot, H. Jarodzka, M. F. van der Schaaf, I. C. van der Schaaf, J. P. J. van Schaik, Th. J. ten Cate

**Affiliations:** 1 Radiology Department, University Medical Center Utrecht, E01.132, Heidelberglaan 100, 3584 CX Utrecht, The Netherlands; 2 Center for Learning Science and Technologies, Open University of the Netherlands, Heerlen, The Netherlands; 3 Department of Education, Utrecht University, Utrecht, The Netherlands; 4 Center for Research and Development of Education, University Medical Center Utrecht, Utrecht, The Netherlands

**Keywords:** Eye tracking, Image interpretation, Medical education, Radiology, Search patterns, Visual diagnosis

## Abstract

Eye tracking research has been conducted for decades to gain understanding of visual diagnosis such as in radiology. For educational purposes, it is important to identify visual search patterns that are related to high perceptual performance and to identify effective teaching strategies. This review of eye-tracking literature in the radiology domain aims to identify visual search patterns associated with high perceptual performance. Databases PubMed, EMBASE, ERIC, PsycINFO, Scopus and Web of Science were searched using ‘visual perception’ OR ‘eye tracking’ AND ‘radiology’ and synonyms. Two authors independently screened search results and included eye tracking studies concerning visual skills in radiology published between January 1, 1994 and July 31, 2015. Two authors independently assessed study quality with the Medical Education Research Study Quality Instrument, and extracted study data with respect to design, participant and task characteristics, and variables. A thematic analysis was conducted to extract and arrange study results, and a textual narrative synthesis was applied for data integration and interpretation. The search resulted in 22 relevant full-text articles. Thematic analysis resulted in six themes that informed the relation between visual search and level of expertise: (1) time on task, (2) eye movement characteristics of experts, (3) differences in visual attention, (4) visual search patterns, (5) search patterns in cross sectional stack imaging, and (6) teaching visual search strategies. Expert search was found to be characterized by a global-focal search pattern, which represents an initial global impression, followed by a detailed, focal search-to-find mode. Specific task-related search patterns, like drilling through CT scans and systematic search in chest X-rays, were found to be related to high expert levels. One study investigated teaching of visual search strategies, and did not find a significant effect on perceptual performance. Eye tracking literature in radiology indicates several search patterns are related to high levels of expertise, but teaching novices to search as an expert may not be effective. Experimental research is needed to find out which search strategies can improve image perception in learners.

## Introduction

An important part of clinical reasoning is based on visual information. Physicians use visual information derived from direct observation of patients (e.g. dermatologic findings) and from other means such as electrocardiograms, histopathology and radiological images. Eye tracking can reflect physicians’ attention with objective measures and provide insight into clinical reasoning processes (Holmqvist et al. 2011). Visual diagnosis plays a central role in diagnostic radiology and eye tracking procedures have proven to be a valuable tool for investigating the visual diagnostic process in radiology for decades (Krupinski 2010).

Several investigators have proposed models that attempt to capture the complexity of radiologists’ visual search. In 1978, Nodine and Kundel proposed a 3-phase theory of visual search and detection, distinguishing initial overall pattern recognition, focal attention for image detail and final decision making (Nodine and Kundel 1987). The first phase refers to the first glance at the image, in which observers compare an anatomic mind map with the perceived image and localize perturbations. Evidence of this holistic search derives from studies in which expert radiologists were found to be able to correctly identify abnormal images within approximately one-fourth of a second (Kundel and Nodine 1975; Carmody et al. 1981; Oestmann et al. 1988). In the following scanning phase, observers examine potential targets and perturbations through fixation (i.e., directing their gaze and visual attention to a specific location). In the third phase, a decision about the presence of a lesion is made, characterized by prolonged or multiple clustered fixations.

More recent research discerns only two components of visual search, embracing a similar concept of a global-focal search model: (1) a relatively fast global impression that signals possible abnormalities and (2) a slower, more detailed focal search for recognition and evaluation of abnormalities. These two components of visual search are defined either as a fast holistic and slow search-to-find mode (Kundel et al. 2007); a pre-attentive filter and cognitive evaluation stage (Swensson 1980); or nonselective and selective pathways running in parallel (Drew et al. 2013a). The concept also aligns with the system 1 and system 2 thinking modes described by Kahneman (2003). The fast, holistic impression of a global search relates to system 1 thinking, which is an automatic and relatively quick thinking process, while the slower focal search mode can be associated with the more attentional and effortful mental activity of system 2 thinking.

These models were developed for static 2D images and may not apply to cross sectional stack imaging where visual search patterns are even more complex. Cross sectional images, such as computed tomography (CT) and magnetic resonance (MR) scans, are currently viewed in stack mode which involves scrolling through a large set of consecutive cross sections of a body region. Searching a stack of cross sectional images differs fundamentally from searching a single image, because the identification and interpretation of abnormalities requires scrolling through a set of images and the visual information changes continuously depending on the level of the cross section.

Perceptual errors, i.e. errors in the detection of abnormalities, account for a major part of misdiagnoses in radiology (Donald and Barnard 2012), and can result from cognitive biases (Jager et al. 2014) or a faulty visual search (Kundel et al. 1978). It is important to identify visual search patterns that do or do not lead to accurate perception of lesions on radiological images. From an educational perspective, the ultimate goal of eye-tracking research in medical image perception is to improve image interpretation by avoiding errors in visual search. When we understand the perceptual process, we may identify search strategies that lead to improved performance of clinicians and integrate these in radiology training. The aim of this review is to identify visual search characteristics that may lead to higher perceptual accuracy. The central question in this review is:

How do visual search characteristics relate to diagnostic performance in radiology?

We explored eye tracking research in the radiology expertise literature to address this question.

To understand this research, some background information is needed about eye movement parameters and how they relate to the global-focal search models. Eye movement parameters that are frequently used in radiological image perception are: *time to first fixation of the abnormality, fixation durations on relevant or redundant areas, number of fixations on relevant or redundant areas, saccades and image coverage.* A *fixation* is a period of time wherein the eye remains still. It reflects attention to that particular area in the image and actual intake of information. *Saccades* are the rapid eye movements in between fixations to re-allocate the focus of attention from one area to another. During a saccade, no visual information can be processed (Holmqvist et al. 2011).

According to the global-focal search models (Swensson 1980; Kundel et al. 2007; Drew et al. 2013a), experts exhibit an efficient search by selecting potential lesions with a global search, taking advantage of a larger functional visual field than novices in combination with a greater conceptual knowledge about where to best look for abnormalities. The global search guides the eyes to suspicious areas for a more detailed focal search. In an effective global search, perturbations are localized fast and *time to first fixation of the abnormality* is expected to be shorter in experts than in less proficient observers (Gegenfurtner et al. 2011). The efficient selection process enables experts to take in all relevant information through fewer fixations, so that *image coverage*, based on location of fixations, may be relatively less in experts’ searches. Consequently, required search time also decreases with increasing expertise. *Saccades* are presumed to enlarge with experience due to an increased visual span (Gegenfurtner et al. 2011; Bertram et al. 2013). An effective global search could decrease the need to fixate intensively on a lesion and could decrease *fixation duration* and *number of fixation* parameters (Gegenfurtner et al. 2011). On the other hand, an ineffective global search can prevent an observer from recognizing the abnormality at all, also decreasing the duration and number of fixations on a lesion. These parameters are probably task-specific (Gegenfurtner et al. 2011; Bertram et al. 2013), so we expect variable relationships to expert level.

In addition to the traditional eye movement parameters, various derivative parameters are increasingly being used to capture and categorize complex search patterns that result from a combination of subsequent eye movements. In this article, we will refer to these parameters as ‘visual search patterns’.

## Materials and methods

A narrative systematic review (Kastner et al. 2012) was conducted. The search strategy was systematic, with a broad sensitive approach. The data of the included studies did not allow for a meta-analysis, due to large variations in methodology, variables and outcome measures. A thematic analysis approach (Mays et al. 2005) was used to extract and arrange the data. Next, results within each theme were integrated and interpreted with a textual narrative synthesis (Lucas et al. 2007), visualizing the differences and similarities in results among varying study methodologies.

### Literature review protocol

**Fig. 1:**
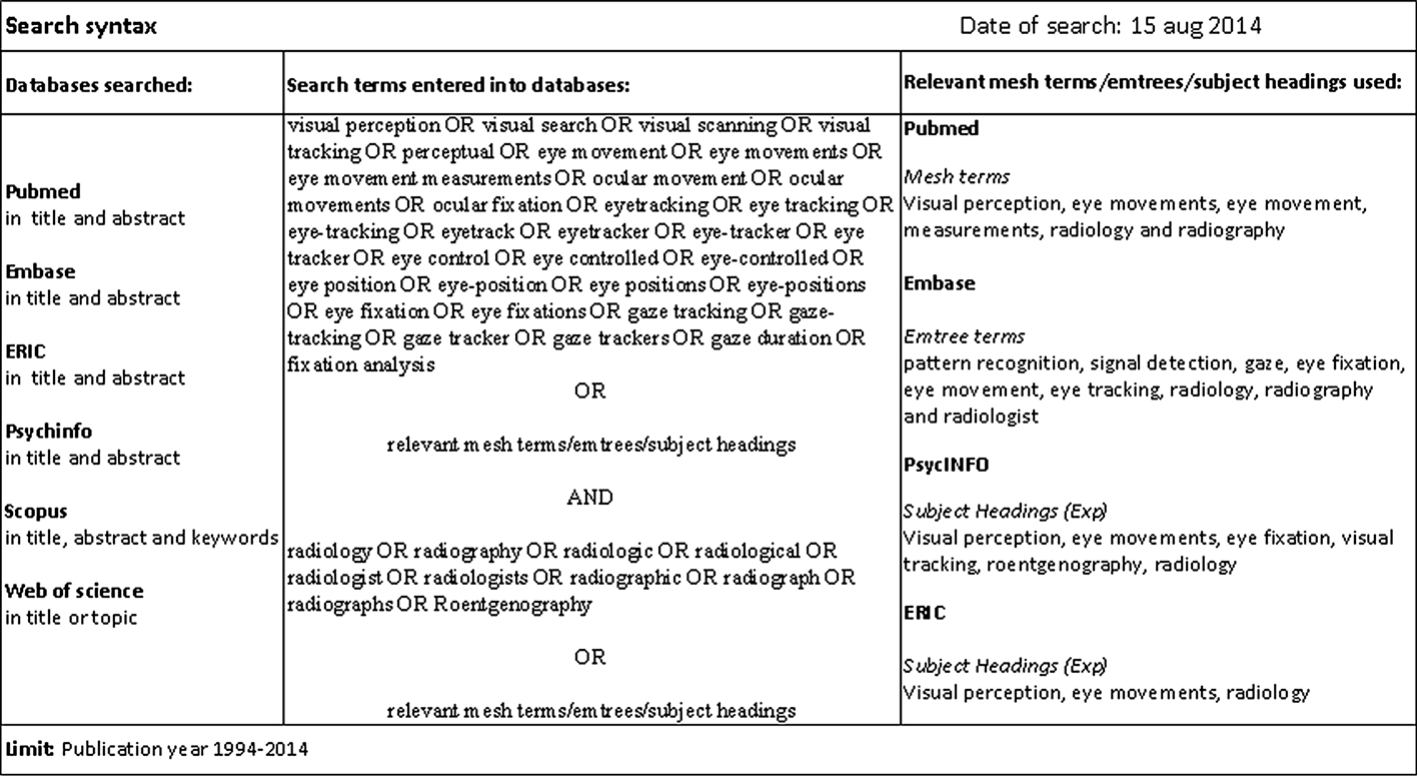
Search syntax

We searched the databases PubMed, EMBASE, ERIC, PsycINFO, Scopus and Web of Science on August 15, 2014. A librarian assisted with the electronic search. The search terms were synonyms of ‘visual perception’ and ‘eye tracking’, combined with the Boolean operator AND to synonyms of radiology. The search strategy consisted of keywords and controlled vocabulary (Fig. 1). Because radiology imaging has tremendously changed in the last decades and only fairly recent literature can reflect current radiology practice, we limited the search to the last 20 years. The search was repeated on July 31, 2015.

### Study eligibility and selection

**Fig. 2:**
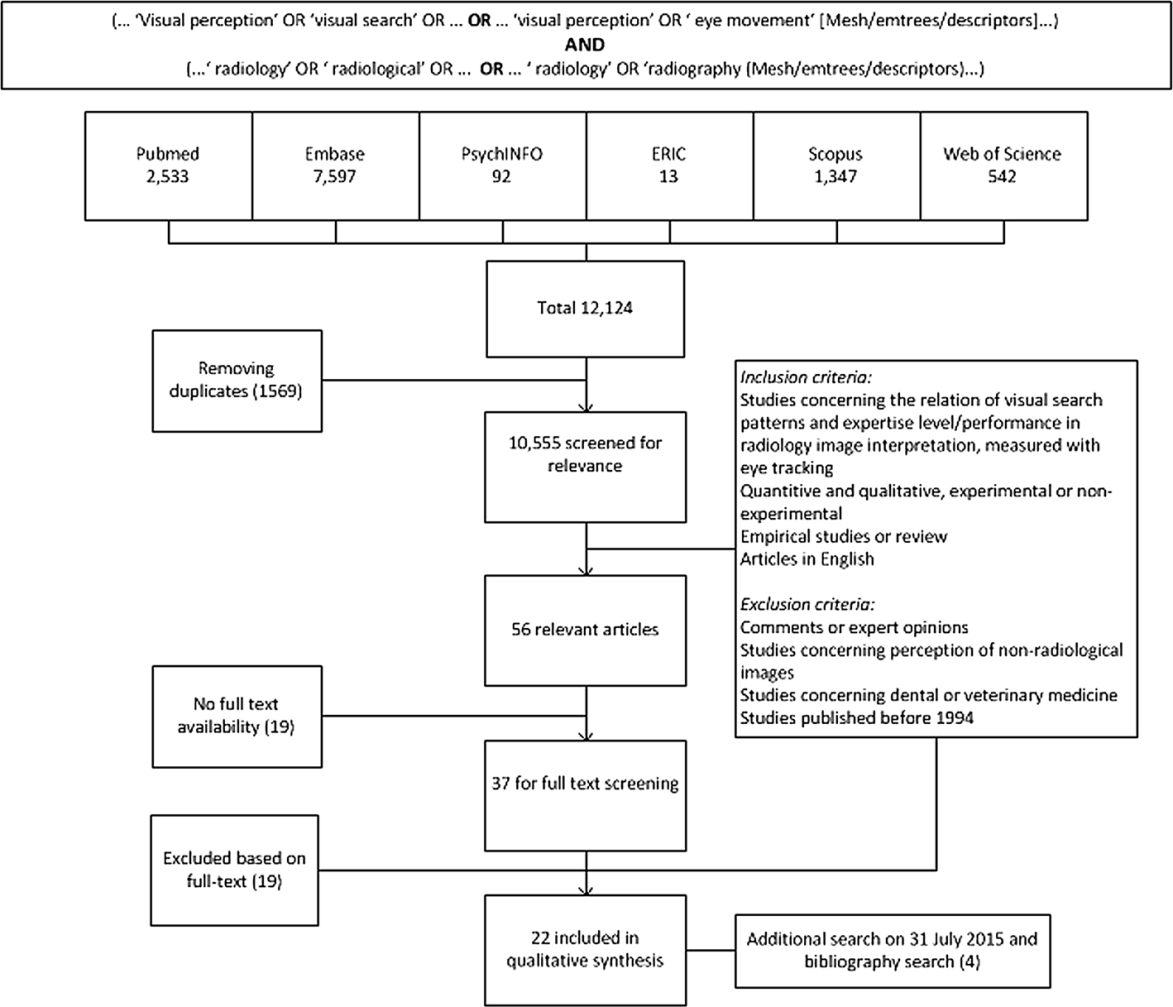
Search flow chart

We included empirical studies published between January 1, 1994 and July 13, 2015. Studies had to address at least one of the three following topics by means of eye-tracking research: (1) the relationship between level of expertise and visual search characteristics, (2) the relationship between performance and visual search characteristics, or (3) the effect of teaching visual search strategies on performance. Reviews were only included for bibliography search. Inclusion and exclusion criteria are summarized in the search flowchart (Fig. 2).

After removing duplicate articles, two authors (C.J.R and A.G.) independently screened titles and abstracts for relevance. Inclusion and exclusion criteria were applied to all potentially relevant full text articles. In case of discrepancy between the two researchers, the eligibility of the study was discussed to reach consensus. If two similar articles were published based on overlapping study data, only the one with the largest sample was included. The bibliographies of included articles were screened for additional relevant articles.

### Data collection, quality assessment

Two reviewers independently extracted characteristics and outcomes of the studies that met the inclusion criteria. Discrepancy between the researchers was discussed until consensus was reached. The quality of the studies was assessed with the Medical Education Research Study Quality Instrument (MERSQI) score (Reed et al. 2007).

### Data analysis and synthesis

The relationship between visual search characteristics and level of expertise was approached in several ways. We could identify six topics for thematic analysis: (1) time on task, (2) eye movement characteristics of experts, (3) differences in visual attention, (4) visual search patterns, (5) search patterns in cross sectional stack imaging, and (6) teaching visual search strategies. All data were categorized in one of these themes. One single study could address multiple themes. For the textual narrative synthesis, the results within each theme were pooled in different subgroups to discover patterns of differences and similarities in results in between or within subgroups. Subgroup results were compared based on differences in task, target, image modality and lesion subtlety (Table 2); similarities and differences in subgroup results were reported.

## Results

The primary search on August 15, 2014 yielded 12,125 articles; after removing duplicates 10,555 remained. Narrowing the search strategy was considered, but key words broadening the search (e.g. visual perception, eye movements) were essential for the research questions and could not be omitted. Titles were screened on relevance. Applying inclusion and exclusion criteria on abstracts of potentially relevant studies resulted in 56 relevant abstracts, of which 37 articles could be retrieved in full-text. After applying inclusion and exclusion criteria on full-text articles, 18 relevant studies remained. Four additional articles were added from the second search on July 31, 2015. Screening reference lists of all included articles and six review articles (Norman et al. 1992; Taylor 2007; Krupinski 2010; Gegenfurtner et al. 2011; Drew et al. 2013a; Blondon et al. 2015) did not provide additional relevant articles. Search results are summarized in Fig. 2.

### Characteristics of included studies

All included studies contained quantitative data. Four studies additionally analyzed qualitative data with respect to the research question (Hu et al. 1994; Krupinski 1996; Alzubaidi et al. 2009; Matsumoto et al. 2011). In total, 526 observers participated in the studies. Quality assessment yielded a mean MERSQI score of 11.5 (out of 18), ranging from 8.5 to 14.5. A major limitation of most studies was the study design. Almost all studies were cross-sectional studies, investigating associations between level of expertise and visual search characteristics, which may have been influenced by many co-varying factors. Only one study investigated the effect of visual search strategies on performance with a randomized controlled trial (Kok et al. 2016). Triangulation of eye tracking data with other measures is important for a sound interpretation of underlying cognitive processes (Kok and Jarodzka 2016). Unfortunately, the vast majority of the studies only related the eye tracking data to performance data and did not triangulate results with verbal data. Sampling was generally done from one institution, except for one study that sampled two institutions (Drew et al. 2013b). The validity evidence for the evaluation instruments varied between studies. For example, several studies did not report any properties of the applied eye-tracking system and whether or not image findings were validated by an expert panel. Overall, strengths of the studies were the objectivity of the data and outcome measures.

The included studies are listed in Table 1. Detailed characteristics of the studies are shown in the “Appendix”. The results of the thematic analysis are listed in Table 2.

**Table 1:** Characteristics and quality assessment of included studies

Publication year	First author	Type of article	Specialty	Design				Variables			
				Correlational	Causal-comparative	Randomized trial	Qualitative components	1. Level of expertise	2. eye tracking measure(s)	3. Retrospective reporting	4. performance
2009	Alzubaidi, M.^a^	CONF	RAD	x			x	x	x	x	
2010	Alzubaidi, M.^b^	CONF	RAD	x				x	x	x	
2013	Bertram, R.	JOUR	RAD		x			x	x		x
2010	Cooper, L.	CONF	RAD		x			x	x		x
2015	Diaz, I.	JOUR	RAD		x			x	x		x
2013	Donovan, T.	JOUR	RAD		x			x	x		x
2013	Drew, T.	JOUR	RAD		x				x		x
2015	Giovinco N.A.	JOUR	ORT		x			x	x		
1994	Hu, C. H.	JOUR	RAD		x		x	x	x		x
2012	Kok, E.^a^	JOUR	RAD		x			x	x		x
2016	Kok, E.^b^ - exp.1	JOUR	RAD		x			x	x		x
2015	Kok, E.^b^ - exp.2	JOUR	RAD			x			x		x
1996	Krupinski, E. A.	JOUR	RAD		x		x	x	x		x
2007	Kundel, H. L.	JOUR	RAD	x					x		x
2007	Leong, J. J. H.	JOUR	ORT/RAD/A&E		x			x	x		x
2014	Mallet, S.	JOUR	RAD		x			x	x		x
2006	Manning, D.^b^	JOUR	RAD		x			x	x		x
2011	Matsumoto, H.	JOUR	NEU		x		x	x	x		
1996	Nodine, C. F.^a^	JOUR	RAD		x			x	x		x
2002	Nodine, C. F.^b^	JOUR	RAD		x			x	x		x
2015	Rubin, G.D.	JOUR	RAD	x					x		x
2013	Voisin, S.	CONF	RAD	x					x		x
2012	Wood, G.	JOUR	RAD	x	x			x	x		x

Publication year	First author	Type of article	Specialty	Quality assessment (MERSQI)						
				Study design	Sampling	Type of data	Validity of evaluation instrument	Data analysis	Outcomes	Total
2009	Alzubaidi, M.^a^	CONF	RAD	1	1	3	0	2	1.5	8.5
2010	Alzubaidi, M.^b^	CONF	RAD	1	1	3	1	2	1.5	9.5
2013	Bertram, R.	JOUR	RAD	1	1	3	2	3	1.5	11.5
2010	Cooper, L.	CONF	RAD	1	1	3	3	3	1.5	12.5
2015	Diaz, I.	JOUR	RAD	1	1	3	1	1	1.5	8.5
2013	Donovan, T.	JOUR	RAD	1	1	3	3	3	1.5	12.5
2013	Drew, T.	JOUR	RAD	1	2	3	3	3	1.5	13.5
2015	Giovinco N.A.	JOUR	ORT	1	1	3	2	2	1.5	10.5
1994	Hu, C. H.	JOUR	RAD	1	1	3	3	3	1.5	12.5
2012	Kok, E.^a^	JOUR	RAD	1	1	3	3	3	1.5	12.5
2016	Kok, E.^b^ - exp.1	JOUR	RAD	1	1	3	3	3	1.5	12.5
2016	Kok, E.^b^ - exp.2	JOUR	RAD	3	1	3	3	3	1.5	14.5
1996	Krupinski, E. A.	JOUR	RAD	1	1	3	3	3	1.5	12.5
2007	Kundel, H. L.	JOUR	RAD	1	1	3	3	3	1.5	12.5
2007	Leong, J. J. H.	JOUR	ORT/RAD/A&E	1	1	3	2	3	1.5	11.5
2014	Mallet, S.	JOUR	RAD	1	1	3	3	2	1.5	11.5
2006	Manning, D.^b^	JOUR	RAD	1	1	3	1	3	1.5	10.5
2011	Matsumoto, H.	JOUR	NEU	1	1	3	2	3	1.5	11.5
1996	Nodine, C. F.^a^	JOUR	RAD	1	1	3	0	3	1.5	9.5
2002	Nodine, C. F.^b^	JOUR	RAD	1	1	3	2	3	1.5	11.5
2015	Rubin, G.D.	JOUR	RAD	1	1	3	3	3	1.5	12.5
2013	Voisin, S.	CONF	RAD	1	1	3	2	2	1.5	10.5
2012	Wood, G.	JOUR	RAD	1	1	3	3	3	1.5	12.5

Research question categories: (1) expert level related to visual search patterns; (2) visual search patterns related to performance; (3) visual search patterns or expert level related to error types

*CONF* Conference paper, *JOUR* Journal article, *RAD* radiology, *ORT* orthopedics, *A&E* accident and emergency, *MERSQI* Medical Education Research Study Quality Instrument, *Exp.* experiment

**Table 2:** Results of thematic analysis

First author	Participants		Type of task			Cases		Themes					
	Total participants	Expert level comparison	Task	Target	Image modality	Total	Lesion subtlety	1. Time on task	2. Eye movement characteristics	3. Differences in visual attention	4. Visual search patterns	5. Search patterns in stack imaging	6. Teaching visual search strategies
Alzubaidi, M.^a^	5	S–E	D + I	Chest lesions	XR	20	NS	X	X		X		
Alzubaidi, M.^b^	5	S–E	D + I	Chest lesions	XR	20	NS				X		
Bertram, R.	38	E–N–L	D	Abdominal lesions	sCT	9	M		X	X			
Cooper, L.	28	E–I–L	D	Stroke	sCT/MR	48	M	X	X	X			
Diaz, I.	6	E–N	D	Lung nodules	sCT	NS	M					X	
Donovan, T.	40	E–N–L	D	Lung nodules	XR	30	S	X		X			
Drew, T.	25	E	D	Lung nodules	sCT	5	S		X			X	
Giovinco, N.A.	16	E–I	I	Bunion	XR	25	NA	X	X				
Hu, C. H.	15	S–E–I–N	D	Lung nodules/fractures	XR	10	S				X		
Kok, E.^a^	30	E–I–N	D + I	Chest lesions	XR	24	S	X	X		X		
Kok, E.^b^—exp.1	20	E–I–N	D + I	Chest lesions	XR	5	NA	X	X		X		
Kok, E.^b^—exp.2	75	N	D + I	Chest lesions	XR	22	M						X
Krupinski, E. A.	6	S–I	D	Breast cancer	XR	20	M	X	X		X		
Kundel, H. L.	9	S–E–I	D + I	Breast cancer	XR	40	S		X		X		
Leong, J. J. H.	25	E–I	D	Fractures	XR	33	NS	X		X	X		
Mallet, S.	65	S–E	D	Colon polyps	CTC	23	M	X	X				
Manning, D.^b^	21	E–N	D	Lung nodules	XR	120	M	X	X				
Matsumoto, H.	24	E–N	D + I	Stroke	tCT	6	M			X			
Nodine, C. F.^a^	15	S–I–N–L	D + I	Breast cancer	XR	9	NS		X				
Nodine, C. F.^b^	9	S–EvI	D + I	Breast cancer	XR	40	S		X	X			
Rubin, G.D.	13	S–E–I	D	Lung nodules	sCT	40	S	X	X				
Voisin, S.	6	S–I	I	Breast masses	XR	40	NA	X	X				
Wood, G.	30	E–I–N	D + I	Fractures	XR	9	M	X	X	X			

*S* subspecialized experts, *E* experts, *I* intermediates, *N* novices, *L* lay people

*D* detection, *I* interpretation

*XR* X-rays, *sCT* stack mode computed tomography, *tCT* tiled mode computed tomography, *MR* magnetic resonance

*S* subtle lesions, *M* mixed subtle and non-subtle, *NA* not applicable, *NS * not specified

1, 2 or 3 = eye tracking measures related to research question 1, 2 or 3

*exp.* experiment

### Time on task

In general visual search time decreases with increasing levels of expertise (Krupinski 1996; Manning et al. 2006; Alzubaidi et al. 2009; Cooper et al. 2010; Kok et al. 2012, 2016; Voisin et al. 2013; Wood et al. 2013; Giovinco et al. 2014; Rubin et al. 2015). The average time experts took to view an image varied largely between studies, ranging from 4 s up to around 45 s, due to differences in task characteristics (detection, interpretation or both; lesion subtlety) and time limits. Novices or lay persons took approximately 1.5–2.5 times longer. In some studies viewing time did not significantly differ between expert levels (Leong et al. 2007; Donovan and Litchfield 2013; Mallett et al. 2014).

### Eye movement characteristics of experts

Studies show a high variance in type of tasks, relating to detection or interpretation, subtlety and number of lesions, image modality and time limits. Despite these differences, there are some consistent patterns in how experts move their eyes compared to less proficient observers: experts fixate faster on an abnormality (Krupinski 1996; Nodine et al. 1996; Kundel et al. 2007; Cooper et al. 2010; Wood et al. 2013; Mallett et al. 2014) and make more fixations (Manning et al. 2006; Alzubaidi et al. 2009; Voisin et al. 2013; Giovinco et al. 2014). Experts or subspecialized experts tended to fixate on an abnormality within 0.5–2 s in mammography and CT studies, around 3 s in skeletal and chest X-rays, and up to 5 s in subtle fracture cases. Novices typically took around 1.5 to two times longer to fixate on a lesion, and up to 4.5 times longer in cases with subtle abnormalities or a second abnormality. Most studies found no significant differences in total fixation duration between expert levels (Kok et al. 2012; Bertram et al. 2013; Giovinco et al. 2014).

Other findings were less consistent across studies. Saccade length was found to be larger in experts than novices in two studies (Manning et al. 2006; Kok et al. 2012), but smaller in one study (Bertram et al. 2013). The short saccades of experts were found in CT scans with visceral abnormalities (e.g. cancer) and related enlarged lymph nodes, which are often grouped and in the vicinity of the visceral abnormality. Variable results were found concerning image coverage: two chest X-ray studies found a negative relation between image coverage and expertise level (Manning et al. 2006; Kok et al. 2016), while two chest CT studies found a positive relationship between image coverage and performance (Drew et al. 2013b; Rubin et al. 2015). Eye movement characteristics of experts are summarized in Table 3.

**Table 3:** The relation between level of expertise and eye tracking parameters

Eye tracking parameters	Association with high levels of expertise (number of studies*)
Total time	Decrease (10)
Time to first fixation	Decrease (6)
Total fixation duration	–
Fixation duration on AOI	Increase (3) or decrease (3)
Dwell time ratio	Increase (1) or decrease (1)
Total number of fixations	Decrease (4)
Number of fixations on AOI	Increase (1) or decrease (1)
Saccade length	Increase (2) or decrease (1)
Image coverage	Decrease (2) or increase (2)

* Only significant results are included

– = No significant difference reported

### Differences in visual attention

Apart from describing pure eye movement characteristics of experts, examining which areas in the image receive more or less attention can give additional insight into the nature of visual expertise. For example, do experts pay more attention to the abnormality (the area of interest, AOI), or do they spend more time on the rest of the image to check for other abnormalities? Results were found to differ across tasks: fixation duration on AOIs decreased in higher level experts in detection only tasks (Leong et al. 2007; Cooper et al. 2010; Donovan and Litchfield 2013), but increased in tasks combining detection and interpretation (Nodine et al. 2002; Matsumoto et al. 2011; Wood et al. 2013). The increase in fixation duration was most prominent in subtle lesions (Matsumoto et al. 2011; Wood et al. 2013). The effect of expertise on the frequency of fixations on a relevant area also differed across tasks. During lung nodule detection, experts fixated less frequently on nodules than novices did (Donovan and Litchfield 2013). In contrast to novices, experts did not increase their fixation frequency, when hydronephrosis (i.e. a dilated renal collecting system) was visible on CT (Bertram et al. 2013). However, when searching CT scans for enlarged lymph nodes, experts fixated more frequently than novices did on a region were enlarged lymph nodes prevail (Bertram et al. 2013). When lymph nodes were present, they further increased their fixation frequency in contrast to novices (Bertram et al. 2013). This difference may be explained by the nature of the tasks. The experts probably did not need as many fixations as the novices to identify a lung nodule or hydronephrosis. They instead deliberately sampled other areas where abnormalities frequently are expected, and increased their attention to assess lymph nodes for enlargement.

Compared to experts, novices are found to give more attention to salient structures, regardless of their relevance. For example, the heart is a salient though relatively unimportant structure on a chest X-ray, because there is generally not much to report about it apart from its size. Naïve observers spend much time on the heart, while experts spend a large percentage of their time on the lungs (Donovan and Litchfield 2013). Similarly, Matsumoto et al. (2011) found that novices fixated more or equally often on salient features in brain CT scans (e.g. physiological calcifications), while experts fixated more on non-salient, but relevant areas, such as the brain parenchyma and inconspicuous lesions.

### Visual search patterns

Several studies distinguished different visual search patterns among different types of tasks (Table 4). Hu et al. (1994) described four types of search patterns (circular, radial, zigzag and complex) in wrist and hand X-ray search and found that experts predominantly follow a radial pattern, i.e. following the digits out and back from the carpus, while novices’ search patterns were more variable. Krupinski (1996) reported that, in mammograms with more than one lesion, after detection of the first lesion, intermediates generally followed a circumferential search pattern, while experts directly proceeded to the second (and third) lesion. Also, experts showed more comparison scanning between the left and right breast. Kok et al. (2012) found a higher global/local ratio in experts viewing normal chest X-ray images, meaning that experts’ visual search patterns were more diffuse than novices. In abnormal images no significant global/local ratio differences were found between expert levels. This research group also found that experts searched normal chest X-rays more systematically than novices (Kok et al. 2016).

**Table 4:** Characteristics of visual search patterns associated with high levels of expertise

Image modality	Visual search patterns in high expert levels
Wrist and hand X-rays	Radial pattern; global-focal pattern
Mammography	Comparison scanning between left and right
	Global-focal pattern
Chest X-rays	Systematic search
	Global-focal pattern
	Diffuse pattern
Chest CT in stack mode	Drilling (scrolling up and down while focusing on one area)

#### Global-focal search patterns

Some studies found quantitative or qualitative evidence that the visual search of experts is characterized by a global-focal search pattern. Quantitative evidence was based on distance to target measures and showed that experts’ search was consistent with a two-stage search pattern: (1) spending time on or near the abnormality, interpreted as time for identification and decision making and (2) spending time relatively far from the abnormality, for cross-referencing or identification of other abnormalities (Leong et al. 2007). The use of a two-stage search was corroborated by qualitative data from another study where radiologists were interviewed following an eye-tracking experiment (Alzubaidi et al. 2009). The most experienced radiologists described a two-step search strategy, starting with holistic perception, followed by more spatially focused visual perception analyzing areas of the X-ray image in detail. In contrast, the radiologist with the least experience described his search strategy as a sequential examination of small areas, a predominantly focal search pattern which was supported by his eye movements (Alzubaidi et al. 2009). Qualitative evidence for a global-focal search pattern was also found in mammography: the best observers typically jumped directly to the malignancy at the beginning of their search, followed by a circumferential scan of the view, and consecutively a long saccade to the malignancy at the second view, again followed by a circumferential scan (Kundel et al. 2007). Alzubaidi et al. (2010) found that less experienced observers fixate more often than experienced observers within one region repetitively, again reflecting a focal search pattern in the less experienced.

### Search patterns in cross sectional stack imaging

Two studies combined eye tracking and scroll behavior data and distilled different search patterns used in cross sectional stack image viewing. Drew et al. (2013b) distinguished two visual search patterns in lung nodule detection in a stack of CT images: scanners and drillers. Scanners tend to visually search a single slide, before scrolling further through the stack, while drillers focus their eyes on one quadrant of the lung field and quickly scroll through the stack in depth before moving to another quadrant. Scanners’ search patterns are characterized by longer saccades and more quadrant fixation clusters compared to drillers. Drillers reached larger lung coverage and identified more pulmonary nodules than scanners. As a co-varying factor, drillers tended to have more experience in searching through chest CT scans than scanners. A second, descriptive study of radiologists’ approach to viewing CT scans (Diaz et al. 2015) found a search approach similar to drilling: an organized way of scrolling and viewing through CT scans by typically scrolling up and down through a quadrant once before moving to a next quadrant. Lay people’s search patterns showed more frequent changes in scrolling and viewing direction and varied more than expert patterns.

### Teaching visual search strategies

Only one study addressed the effect of teaching specific search strategies and compared three visual search trainings in chest X-ray viewing: systematic viewing, non-systematic viewing and full coverage viewing. The systematic viewing training led to a more systematic search and the full coverage training to a larger image coverage, but neither resulted in higher diagnostic performance compared to the non-systematic viewing group. The full coverage group even performed worse than the non-systematic viewing group (Kok et al. 2016).

## Discussion

The eye-tracking literature of the past 20 years details the relationship between visual search characteristics and level of expertise within six main themes: time on task, eye movement characteristics of experts, differences in visual attention, visual search patterns, search patterns in cross sectional stack imaging, and the effect of teaching visual search strategies. Studies varied considerably with respect to multiple study characteristics, such as expertise levels, type of tasks, eye-tracking parameters and performance measures. However, some consistent results were found across studies and in general supported the global-focal search theory. Experts and high performers consistently need shorter viewing times and fixate on abnormalities faster than less experienced readers or low performers. Experts needed up to 5 s to first fixate on subtle abnormalities. From previous non-eye-tracking studies we know that experts can make correct diagnostic decisions based on much shorter image exposures, even in exposures as short as 200 ms (Kundel and Nodine 1975). Such short time spans do not allow for searching the image and fixating on abnormalities; rather abnormalities are probably perceived through taking advantage of their large visual span. Different from most of the cases reported in our review, the cases used in the 200 ms study of Kundel et al. were large or diffuse abnormalities that can be assessed more globally than subtler findings.

Findings regarding number of fixations and saccade length also supported the global-focal search theory. Number of fixations decreased with higher expertise levels, probably because experts need less fixations to collect relevant image information (Gegenfurtner et al. 2011). This also aligns with shorter viewing times needed by experts. Saccade length was found to be generally larger in experts than novices, which aligns with an effective global search, because large saccades towards a disturbed signal are likely to follow a successful global search (Kundel et al. 2007). However, experts were found to shorten their saccades in CT scans with grouped enlarged lymph nodes that were related to and in proximity of visceral abnormalities, probably because they *suspected* the enlarged lymph nodes to be in proximity of the detected visceral abnormality and near each other, based on knowledge and experience. This underscores that search patterns of experts are driven by knowledge and that they anticipate their search based on what they have already found.

Differences in visual attention across levels of expertise revealed that experts tend to focus on relevant though not necessarily salient structures, whereas novices tend to look at salient structures regardless of their relevance. We found some inconsistent results across studies with respect to differences in visual attention to abnormalities, though this may be explained by differences in type of task. Experts spend less time fixating on lesions than novices in detection only tasks, probably because they make fast normal–abnormal decisions and quickly proceed searching the rest of the image for other findings. When diagnostic reasoning is added to the task, experts spend more time on the lesion than novices. Because this difference was most pronounced in subtle or non-salient findings, it may be explained by novices’ failures to recognize the findings as abnormal hindering them from analyzing its features.

Both quantitative and qualitative analyses yielded evidence for a global-focal search pattern that is more prominent in those with higher expertise levels. Quantitative evidence included a fast initial fixation on the abnormality by experts due to developed global searches, and distance to target measures showing that experts adhere to a two-stage search pattern distinguishing searches near and far from the abnormality. Qualitatively, the global-focal search pattern was confirmed by experts in retrospective reports and by sequenced eye-tracking data showing a fast jump towards the abnormality followed by a circumferential scan of the rest of the image. In contrast to experts, novices seem to exhibit a predominantly focal search pattern. Beyond the global-focal search patterns, a wide variety of other search patterns have been explored in the included studies. Some patterns are potentially relevant for teaching purposes, such as the radial search in hand and wrist X-rays, or drilling through chest CT’s. These patterns suggest that expert observers have deliberately adopted such strategies to accomplish a complete and efficient search. However, this is not confirmed by verbal data in the vast majority of studies and the observer’s experience could be tacitly driving the search pattern. Therefore, it is doubtful learners’ perceptual performance will improve upon incorporating the search strategy of experts if they lack experience and knowledge.

Some visually dependent specialties, such as radiology, have witnessed huge technological developments. Only a minority of the studies investigated visual search in volumetric image perception in radiology, possibly because of challenges that are related to eye tracking in such imaging (Venjakob and Mello-Thoms 2015). The large amount of information in volumetric images increases the risk of perceptual errors and introduces particular challenges such as abnormalities that can only be visualized in certain contrast settings (McCreadie and Oliver 2009). Because visual search in cross-sectional image stacks requires image manipulation (van der Gijp et al. 2014) and knowledge and skills needed to interpret stacks of images differ substantially from 2D image interpretation (van der Gijp et al. 2015), evidence for effective search patterns in both visualization modes is not indisputably exchangeable. For example this review shows contrary results with respect to image coverage which can be possibly explained by differences in image modality. In X-ray viewing, experts tended to cover a smaller area of the image than novices did, which can be explained by a larger visual span of experts, but contrary results were found in CT scan reading. In the study of Drew et al. (2013b) high performers who tended to scroll back and forth through the image while fixating on one area (i.e. drillers) showed larger image coverage than lower performers that scanned the complete image slide by slide (i.e. scanners). Searching through cross-sectional image datasets in multiple planes and contrast settings (Andriole et al. 2011) demands a joint effort of the observer’s hands and eyes to visualize the image information. When scanning the complete area of one slide before proceeding to the next slide, large distances have to be bridged with eye movements which is inefficient and may cause skipping of areas. Drilling through images, fixating on one area and scrolling through, requires much less eye movements and more systematically addresses all areas of the image. Because cross-sectional imaging is increasing and contains much visual information (Andriole et al. 2011), finding efficient strategies in different types of cross-sectional search tasks is gaining importance. A recent study investigated zooming behavior in microscope navigation in clinical pathology (Jaarsma et al. 2015). Expertise was characterized by less magnifications. This aligns with the global-focal search pattern; magnification allows for a more detailed search consistent with the focal search-to-find search patterns of novices, versus a more global (zoomed out) view of experts.

Only one eye-tracking study investigated whether training learners to apply expert visual search strategies, in this case systematic search, can improve perceptual performance. Even though the systematic training had a positive effect on the use of systematic viewing, diagnostic performance did not improve. This underscores that we should not assume that teaching expert skills directly to learners is likely to be effective. Much of the evidence in this review supports the global-focal search pattern. But should we teach novices to start with a proficient global search, followed by a focal search? The efficiency of this approach is questionable. The efficient, quick approach of experts is probably driven by hypotheses based on knowledge and experience, in which case it would not make sense to instruct a novice to look at an abnormality more quickly. It may be more beneficial to teach novices where to look in certain clinical circumstances, guided by clinical information or other related findings. Another approach to direct learners` attention to specific areas are Eye Movement Modelling Examples. These are scan paths of experts that can guide learners` visual attention to relevant areas (Jarodzka et al. 2012; Vitak et al. 2012). Other, more defined search strategies mentioned in this review may be relevant to teach to novices, for example drilling though CT scans or radial search in wrist X-rays. But even if we can teach students to search accordingly, will this help them perform better? Randomized trials comparing these search strategies with each other or with free searching are needed to find out which visual search strategies can and should be taught.

Eye-tracking studies in other visual diagnostic professions such as pathology (Jaarsma et al. 2015) and in patient observations for diagnosing neurological disorders (Balslev et al. 2012), show similar evidence for holistic and efficient visual search skills of experts. The holistic search characterized by a fast identification of the abnormality and efficient sampling of the rest of the image aligns with the rapid hypothesis generation and efficient and limited data gathering seen in expert clinical reasoning, attributed to the efficient data processing of System 1 thinking processes (Norman 2009). This is in contrast to the more extensive data gathering in System 2 thinking processes, most apparent in novices who sample the entire image with a focal search pattern (Manning et al. 2006; Alzubaidi et al. 2009; Alzubaidi et al. 2010; Kok et al. 2016). It is obvious that novices’ and experts’ search and thinking processes differ, but for both clinical reasoning and visual search it is unclear which approach is most effective for novices (Norman 2009), or how either approach should be taught.

Some limitations of our study must be reported. Although we did an exhaustive literature search, we may not have captured all literature concerning the relationship between visual search characteristics and perceptual performance. We only included studies from the last 20 years, because technical improvements have had large influence on image quality and representation in the last decades (Andriole et al. 2011). We also confined our search to radiology, and although the results might apply to other visual search tasks, we cannot assure that the results are transferable. Finally, we focused on eye-tracking studies and did not include, for example, think-aloud studies that give more insight on the cognitive aspects of visual search. We reduced a large number of titles to a fairly small number of relevant articles, and relevant articles could have been missed. To reduce this risk, two raters applied clear, objective criteria to exclude articles. Cross referencing did not reveal any missed relevant articles.

In conclusion, visual diagnosis research of the past 20 years has predominantly focused on finding differences between experts and novices, providing clues for improving image perception. Heterogeneity of tasks and outcome measures complicates data synthesis and drawing conclusions. In addition, hardly any studies looked at how to improve image perception by teaching visual search strategies based on these theories. For educational purposes, we should shift from describing differences between experts and novices to defining search strategies and investigating methods to teach novices to search efficiently.

## Appendix

**Table:** Detailed study characteristics, sorted by target

First author	Participants								
	Total participants	Subspecialized experts	Radiologists or other medical specialists	Interns/residents/SHO/registrars	Medical students	Radiographers (technicians)	Student radiographers	Naïve observers	Novices (not specified per group)
Alzubaidi, M.^a^	5	3	2	–	–	–	–	–	–
Alzubaidi, M.^b^	5	3	2	–	–	–	–	–	–
Kok, E.^a^	30	–	9	10	11	–	–	–	–
Kok, E.^b^—exp.1	20	–	9	10	11	–	–	–	–
Kok, E.^b^—exp.2	75	–	–	–	75	–	–	–	–
Diaz	6	–	3	–	–	–	–	3	–
Donovan, T.	40	–	10	–	–	20	–	10	–
Drew, T.	25	–	25	–	–	–	–	–	–
Manning, D.^b^	21	–	8	–	–	5	8	–	–
Rubin, G.D.	13	5	2	6	–	–	–	–	–
Krupinski, E. A.	6	3	–	3	–	–	–	–	–
Kundel, H. L.	9	3	3	3	–	–	–	–	–
Nodine, C. F.^a^	15	3	–	5	–	2	–	5	–
Nodine, C. F.^b^	8	3	3	3	–	–	–	–	–
Voisin, S.	6	2	–	4	–	–	–	–	–
Cooper, L.	28	–	8	10	–	–	–	10	–
Matsumoto, H.	24	–	12	–	–	–	–	–	12
Leong, J. J. H.	25	–	11	14	–	–	–	–	–
Wood, G.	30	–	10	10	–	–	10	–	–
Hu, C. H.	15	3	1	8	3	–	–	–	–
Giovinco, N.A.	16	–	7	–	–	–	–	–	9
Bertram, R.	38	–	7	–	–	9	–	22	–
Mallet, S.	65	27	38	–	–	–	–	–	–

First author	Type of task						Cases			Performance measures	
	Detection task	Detection > 1 lesion	Interpretation task	Time limit	Target	Image modality	Total	Abnormal (%)	Lesion subtlety	TP/FP/TN/FN	Other
Alzubaidi, M.^a^	x	NS	x	–	Chest lesions	XR	20	NS	NS	–	
Alzubaidi, M.^b^	x	NS	x	–	Chest lesions	XR	20	NS	NS	–	
Kok, E.^a^	x	x	x	–	Chest lesions	XR	24	67	S	–	acc
Kok, E.^b^ - exp.1	x	–	x	–	Chest lesions	XR	5	0	NA	TN	
Kok, E.^b^ - exp.2	x	x	x	–	Chest lesions	XR	22	91	M	TP/TN	
Diaz	x	–	–	–	Lung nodules	sCT	NS	NS	M	TP	
Donovan, T.	x	x	–	–	Lung nodules	XR	30	50	S	TP/FP	acc
Drew, T.	x	x	–	x	Lung nodules	sCT	5	100	S	TP/FN	
Manning, D.^b^	x	x	–	x	Lung nodules	XR	120	NS	M	–	AUC
Rubin, G.D.	x	x	–	–	Lung nodules	sCT	40	100	S	TP	
Krupinski, E. A.	x	x	–	–	Breast cancer	XR	20	75	M	TP/TN/FP/FN	
Kundel, H. L.	x	–	x	–	Breast cancer	XR	40	50	S	TP/FN/FP/TN	AUC
Nodine, C. F.^a^	x	NS	x	x	Breast cancer	XR	9	44	NS	TP/FP	d
Nodine, C. F.^b^	x	x	x	–	Breast cancer	XR	40	50	S	TP/FP/FN/TN	AUC
Voisin, S.	–	–	x	NS	Breast masses	XR	40	100	NA	–	
Cooper, L.	x	–	–	–	Stroke	sCT/MR	48	75	M	TP/FP	
Matsumoto, H.	x	–	x	x	Stroke	tCT	6	83	M	–	AUC
Leong, J. J. H.	x	x	–	–	Fractures	XR	33	85	NS	TP/FN	
Wood, G.	x	–	x	–	Fractures	XR	9	67	M	–	acc
Hu, C. H.	x	–	–	x	Lung nodules/fractures	XR	10	70	S	TP/FN/FP	
Giovinco, N.A.	–	–	x	–	Bunion	XR	25	NS	NA	–	
Bertram, R.	x	x	–	NS	Abdominal lesions	sCT	9	67	M	TP/TN	acc
Mallet, S.	x	x	–	x	Colon polyps	CTC	23	100	M	TP	

*SHO* Senior House Officer

*XR* X-rays, *sCT* stack mode computed tomography, *tCT* tiled mode computed tomography, *MR* magnetic resonance

*S* subtle lesions, *M* mixed subtle and non-subtle, *NA* not applicable, *NS* not specified

*TP* true positives, *TN* true negatives, *FP* false positives, *FN* false negatives, *acc* accuracy, *AUC* area under the receiver operating characteristic (ROC) curve, *d* detectability index
